# Evidence of Strong Flux Underestimation by Bulk Parametrizations During Drifting and Blowing Snow

**DOI:** 10.1007/s10546-021-00653-x

**Published:** 2021-08-11

**Authors:** Armin Sigmund, Jérôme Dujardin, Francesco Comola, Varun Sharma, Hendrik Huwald, Daniela Brito Melo, Naohiko Hirasawa, Kouichi Nishimura, Michael Lehning

**Affiliations:** 1 School of Architecture, Civil and Environmental Engineering, Ecole Polytechnique Fédérale de Lausanne, Lausanne, Switzerland; 2 WSL Institute for Snow and Avalanche Research SLF, Davos, Switzerland; 3 National Institute of Polar Research, Tokyo, Japan; 4 Graduate School of Environmental Studies, Nagoya University, Nagoya, Japan

**Keywords:** Eddy-covariance technique, Large-eddy simulations, Monin–Obukhov similarity theory, Snow transport, Turbulent heat fluxes

## Abstract

The influence of drifting and blowing snow on surface mass and energy exchange is difficult to quantify due to limitations in both measurements and models, but is still potentially very important over large areas with seasonal or perennial snow cover. We present a unique set of measurements that make possible the calculation of turbulent moisture, heat, and momentum fluxes during conditions of drifting and blowing snow. From the data, Monin–Obukhov estimation of bulk fluxes is compared to eddy-covariance-derived fluxes. In addition, large-eddy simulations with sublimating particles are used to more completely understand the vertical profiles of the fluxes. For a storm period at the Syowa S17 station in East Antarctica, the bulk parametrization severely underestimates near-surface heat and moisture fluxes. The large-eddy simulations agree with the eddy-covariance fluxes when the measurements are minimally disturbed by the snow particles. We conclude that overall exchange over snow surfaces is much more intense than current models suggest, which has implications for the total mass balance of the Antarctic ice sheet and the cryosphere.

## Introduction

Sublimation of snow and ice is a significant component of the surface mass balance of the Antarctic ice sheet, which is an important driver of sea-level rise. However, current sublimation estimates from large-scale atmospheric models are uncertain because snow transport by wind can strongly amplify sublimation rates, and this effect is not, or is inaccurately, represented in large-scale models (Van Wessem et al. 2018; Agosta et al. 2019).

Even on a local scale, drifting and blowing snow can make the quantification of sublimation challenging. The term drifting snow is used for snow transport close to the surface while blowing snow refers to snow particles transported above a certain height. Here, drifting snow is defined by the transport modes of saltation and creep, which occur mainly in the lowest 0.1 m of the atmosphere. Blowing snow is defined by the transport mode of suspension, roughly corresponding to heights above 0.1 m above the surface.

In situ measurements of sublimation and evaporation often apply the eddy-covariance (EC) method or the bulk parametrization based on Monin–Obukhov similarity theory (MOST). These measurements are not able to capture the part of blowing-snow sublimation that occurs above the measurement height during intense storms. Additionally, snow transport increases the uncertainties of the EC method and the MOST parametrization due to technical and theoretical limitations, respectively. The EC method is affected by advected or falling snow particles or rain droplets which pass through the measurement volume of open-path instruments perturbing the measurement signal (Pomeroy and Essery 1999; Bintanja 2001; LI-COR 2004; Campbell Scientific 2017). This problem is typically visible from spikes in the raw data and sometimes indicated by diagnostic values provided by the instruments. During intense blowing-snow or precipitation periods, the transducer pathways of the sonic anemometer may become obscured, resulting in spurious data or data gaps. While several studies used the EC method at snow-covered sites for validating sublimation estimates or associated parameters (e.g., King and Anderson 1994; Box and Steffen 2001; Stössel et al. 2010; Reba et al. 2012), a few studies discussed the limitations of this technique associated with blowing-snow events. For example, Pomeroy and Essery (1999) mentioned that precipitation and blowing-snow particles near sensor heads perturb the measurements of the sonic anemometer. Nevertheless, they trusted the EC method because the diagnostic indication from the sonic anemometer were acceptable. Bintanja (2001) presented EC measurements during a storm at Svea Station, Drauning Maud Land, Antarctica. Although the diagnostic indication from the sonic anemometer frequently indicated a reduced data quality, turbulence characteristics and spectra were in line with standard surface-layer theory and averaged measurement values were consistent with reference measurements. High sublimation rates with a maximum of $100~\text {W m}^{-2}$ were measured at a height of 2 m during the storm. Nowadays, statistical spike removal is used to minimize the influence of data records affected by precipitation or blowing snow (Vickers and Mahrt 1997; Mauder et al. 2013).

Furthermore, the accuracy of the EC method depends on the validity of a few general assumptions. The method yields the vertical turbulent flux at the measurement height. This flux is only equal to the sum of all sources and sinks of the considered quantity in a representative volume of air between the surface and the measurement height if (i) the mean vertical velocity component is zero (i.e., vertical advection is negligible), (ii) the divergence of horizontal advection of moisture, heat, etc. is zero, (iii) the divergence of the horizontal turbulent flux is zero, and (iv) the conditions are stationary during the averaging period. These assumptions generally require flat and horizontally homogeneous terrain to be fulfilled (Mauder et al. 2010). A violation of these assumptions can lead to a bias in the surface exchange. In sloped terrain, cold-air drainage can result in significant horizontal and vertical advection terms (Leuning et al. 2008). Several studies have indirectly evaluated the assumptions of the EC method by checking the closure of the surface energy balance using additional measurements of the radiation fluxes, the ground heat flux, and changes in heat storage in the volume between the surface and the height of the EC measurements. Wilson et al. (2002) found a general lack of energy-balance closure at many sites in different climates. The terrain at these sites was flat to hilly and, on average, the EC-based exchange of sensible and latent heat was approximately 20% lower than the available energy, i.e., net radiation minus ground heat flux and change in heat storage. Although the imbalance can also be influenced by uncertainties in the radiation fluxes and the ground heat flux or by a mismatch between the footprint areas of different energy fluxes, the frequent lack of energy-balance closure at many sites has caused some concerns regarding limitations of the EC method. For a flat and heterogeneous site, Mauder et al. (2010) identified buoyancy-driven quasi-stationary circulations as a likely reason for an energy imbalance during daytime. In mountainous terrain, a strong violation of the assumptions of the EC method is expected and a pronounced lack of energy-balance closure can be observed (Stiperski and Rotach 2016). Additionally, the EC fluxes can be affected by a loss of high-frequency and low-frequency contributions, although correction procedures exist (Massman 2000; Moncrieff et al. 2004).

The MOST parametrization is based on the same assumptions and additionally assumes that turbulent fluxes are constant with height in the surface layer of the atmosphere (Monin and Obukhov 1954). The latter implies that the exchange of moisture, heat, and momentum between the air and solid or liquid media only occurs at the surface and not within the atmosphere. Snow transport violates this assumption because a significant part of the exchange happens at the surface of drifting/blowing-snow particles. For example, the latent heat flux (*LE*) increases with height in the layer of drifting and blowing snow if the snow particles are net moisture sources at all heights of this layer. In these conditions, the MOST parametrization is expected to underestimate the sum of surface and drifting/blowing-snow sublimation because the parametrization does not account for the large surface area and the strong ventilation of the particle–air interface (Schmidt 1982).

Thiery et al. (2012) and Barral et al. (2014) investigated snow sublimation at Antarctic sites and assumed that the MOST parametrization is still a good estimate of surface sublimation only (i.e., the part of the phase change that happens at the snowpack surface) during periods of drifting snow. However, the MOST parametrization may overestimate surface sublimation but underestimate total sublimation during drifting-snow events if drifting snow represents a net moisture source and a net momentum sink. The former reduces the vertical humidity gradient at the surface and the latter reduces turbulence near the surface, resulting in lower rates of surface sublimation. To properly account for these effects, the local gradients at the surface would be needed, but these are typically not available. If turbulent fluxes vary with height, the result of the MOST parametrization depends on the upper measurement height. In drifting-snow conditions, the MOST parametrization rather reflects a case without drifting snow and with the same humidity and wind speed at the considered heights as in the real drifting-snow case, at least if a constant roughness length is used.

Due to the challenges described above, the current knowledge on the sublimation of drifting and blowing snow is largely based on models. On local scales, large-eddy simulations coupled with a Lagrangian stochastic model (LES–LSM) have become a valuable tool for studying the thermodynamic interactions between particles and the air and to test assumptions made in simpler or larger-scale models (Sharma et al. 2018; Wang et al. 2019). A first quantitative comparison between in situ field measurements and LES–LSM results with respect to the sublimation of drifting and blowing snow was presented by Wang et al. (2019). However, these simulations assumed zero surface fluxes of latent and sensible heat at the lower domain boundary and the comparison did not involve direct turbulence measurements but a parametrization of the sublimation of drifting and blowing snow based on mean wind speed, humidity, and air temperature measurements at several heights (Bintanja 2001). Therefore, further comparisons between sublimation measurements and state-of-the-art numerical models are necessary to constrain sublimation rates in conditions of drifting and blowing snow.

The overarching objective of the present study is to explain differences between the sublimation rates measured using two common in situ methods during snow-transport events. The following hypotheses are tested: (i) the MOST parametrization results in significant errors in turbulent fluxes during snow-transport events due to the false assumption of height-constant fluxes (theory-related errors); (ii) the EC method provides more reliable estimates for turbulent fluxes during snow-transport events, at least if the snow is mainly transported below the measurement height and snowfall is absent. Although the focus is on the latent heat flux (*LE*), the sensible heat flux (*H*) and the momentum flux ($\tau$) are also compared between the measurement techniques. A drifting/blowing-snow event at the S17 site, Queen Maud Land, Antarctica, is discussed in detail. Using a case study with saltation-dominated snow transport and a case study with negligible snow transport, the coherence between the measurements and LES–LSM results is examined and the uncertainties of the MOST parametrization are estimated.

## Methods

### Measurement Site and Instrumentation

**Fig. 1:**
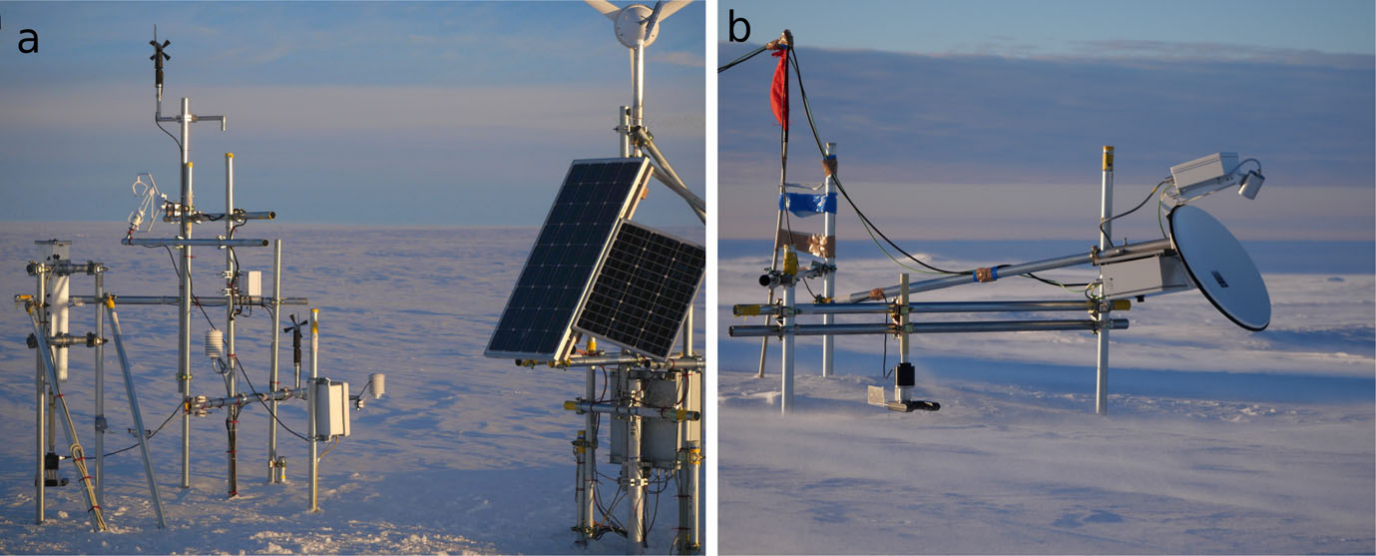
Measurement set-up at the S17 site: **a** micrometeorological station and **b** the MRR and SPC devices

The S17 site ($69^\circ 01^\prime 28^{\prime \prime }\hbox {S}$, $\hbox {40}^\circ 05^\prime 14^{\prime \prime }\hbox {E}$, 600 m above sea level) is located near the Japanese research station Syowa. The surrounding terrain is flat and homogeneous with a slight slope of less than $2^\circ$ towards the coast, which is approximately 15 km west of the S17 site. Apart from some uncovered hills and rocks at the coast, the area is covered by snow throughout the year. During a field trip in January 2019, the wind direction was dominated by north-easterly to south-easterly directions with an unobstructed fetch of hundreds of kilometres over a homogeneous snow surface. This study investigates the period from 10 to 13 January 2019, which includes an intense blowing-snow event. A micrometeorological station was equipped with a three-dimensional ultrasonic anemometer, an open-path infrared gas analyzer, a snow particle counter (SPC), an infrared radiometer for surface-temperature measurements, and standard meteorological sensors (Fig. 1a). Instruments, manufacturers, measurement heights, and data-acquisition intervals are specified in Table 1. Turbulent fluxes were computed using both the EC method and the MOST parametrization. From the ultrasonic anemometer (CSAT3, Campbell Scientific, Logan, USA), the digital output was used, which has a higher resolution than the analogue output.

In addition to the micrometeorological station, a Micro Rain Radar, MRR (MRR-Pro, METEK, Elmshorn, Germany), was installed at a horizontal distance of approximately 500 m. Although this instrument is usually deployed as a vertically pointing radar for precipitation measurements (Peters et al. 2002; Maahn and Kollias 2012), a tilted configuration with an elevation angle of $7^\circ$ was tested to observe the blowing-snow layer. Recently, Walter et al. (2020) demonstrated that a horizontally pointing radar can provide valuable data on blowing snow off a mountain ridge. In the present study, MRR measurements were used to estimate the depth of the blowing-snow layer and to assist in data interpretation. The offset parabolic antenna was installed such that its centre was at a height of 0.8 m and it was facing the dominant wind direction. With a range-gate length of 10 m, the vertical spacing between the range gates was 1.22 m and the measurements ranged up to a height of 39 m. The Doppler spectrum covered radial velocity components between 0 and $48~\text {m s}^{-1}$. The time interval for internal averaging was initially 32 s and was conditionally reduced to 1 s when snow transport became intense. At the location of the MRR, an additional SPC was installed at a height of approximately 0.1 m above the surface.

### Processing of Measurement Data

All measurements were aggregated into 10-min intervals. This time scale was chosen to avoid a significant influence of non-turbulent motions such as gravity waves on the EC turbulent fluxes.

Because a naturally ventilated radiation shield was used with the temperature and relative humidity probe, periods with low wind speeds resulted in an overestimation of the air temperature due to solar heating. This problem was evident from a comparison with the sonic temperature (not shown). Therefore, the air temperature was discarded if the wind speed was below $1~\text {m s}^{-1}$.

**Table 1:** Instruments, variables, measurement heights (*z*), and data-acquisition interval (${\Delta } t$) for the micrometeorological station

Instrument	Instrument model	Variables	*z* (m)	${\Delta } t$ (s)
Ultrasonic anemometer	CSAT3, Campbell Scientific, Logan, USA	Wind velocity components, sonic temperature	1.9	0.05
IR$^\textrm{a}$ gas analyzer	LI-7500, LI-COR, Lincoln, USA	Water-vapour molar density, air pressure	1.9	0.05
Propeller anemometer	Wind monitor 05108-45, Young, Traverse City, USA	Wind speed and direction	1.0, 3.0	30
Temperature/ humidity probe	CS215, Campbell Scientific, Logan, USA	Air temperature, relative humidity	1.0	30
IR$^\textrm{a}$ radiometer	SI-111, Campbell Scientific, Logan, USA	Surface temperature	$0^\textrm{b}$	30
Snow particle counter	SPC-95, Niigata Electric, Japan	Particle number per size class	0.15	1

$$^\textrm{a}$$Infrared

$^\textrm{b}$Nominal height in contrast to the installation height of the radiometer of $z=0.5$ m

#### Eddy-Covariance Method

The EC method enables a direct quantification of vertical turbulent fluxes by means of high-frequency measurements of the respective covariance components. For example, the latent heat flux $(\text {W m}^{-2})$ is given by

1 $$\begin{aligned} LE = L~\overline{w'\rho _v'}~, \end{aligned}$$

where $\overline{w'\rho _v'}~(\text {kg m}^{-2}~\textrm{s}^{-1})$ is the covariance between the vertical velocity component, $w~(\text {m s}^{-1})$, and water-vapour density, $\rho _v~(\text {kg m}^{-3})$, and $L~(\text {J kg}^{-1})$ is the latent heat of sublimation. A positive *LE* indicates an upward flux (sublimation) while a negative *LE* indicates a downward flux (vapour deposition). The same sign convention applies to other turbulent fluxes.

The data post-processing included the removal of artefacts and spikes, a bias correction for vapour density, and common corrections for time lags, for the difference between sonic temperature and air temperature, for density fluctuations, and for spectral losses. Details on these procedures are presented in the Appendix. Block averaging was used to calculate the fluctuations (the ‘prime’ quantities). A large part of the post-processing was performed using the EddyPro$^\circledR$ Software (LI-COR Biosciences 2019). Finally, a quality-control procedure similar to that in Mauder et al. (2013) was applied. The EddyPro$^\circledR$ output includes quality-control results of the tests on steady state and well-developed turbulence described by Foken et al. (2004). Each test yielded a flag of 0, 1, or 2, indicating good, intermediate, or bad quality, respectively. Additionally, a test on missing data (not a number test, NaN test) was introduced, yielding a flag of 0, 1, or 2 if the fraction of missing data was $\le 10$%, between 10% and 25%, or $>25$%, respectively. The flags of the three tests were summed and values higher than 2 were set to 2 to continue the concept of three quality classes. Taking into account that the variables *LE* and *H* depend on each other due to the correction for the difference between the sonic temperature and air temperature and the correction for density fluctuations, the flag of one of these fluxes was increased by $+1$ if the quality of the other flux was bad (Mauder et al. 2013).

#### Monin–Obukhov Bulk Parametrization

According to MOST, the latent heat flux ($\text {W m}^{-2}$) can be expressed as (e.g., Monin and Obukhov 1954; King et al. 2001)

2 $$\begin{aligned} LE = -\rho L C_q~{\bar{u}}~({\bar{q}}_z-{\bar{q}}_0), \end{aligned}$$

where ${\bar{q}}_z$ and ${\bar{q}}_0~(\text {kg kg}^{-1})$ are average specific humidity at height *z* (m) and at the surface, respectively, ${\bar{u}}~(\text {m s}^{-1})$ is average wind speed at height *z*, and $\rho ~(\text {kg m}^{-3})$ is the air density. The specific humidity at the surface was calculated from the measured surface temperature assuming saturation. Although the radiometer measurements of surface temperature are influenced by the temperature of drifting and blowing snow, the resulting bias is expected to be small because the concentration of particles quickly decreases with height and saltating particles are not expected to reach a thermal equilibrium with the air due to their short residence time in the air (Sharma et al. 2018). The dimensionless exchange coefficient for moisture, $C_q$, is given by

3 $$\begin{aligned} C_q = \frac{\kappa ^2}{\left[ \textrm{ln}\left( \frac{z}{z_{0q}} \right) - {\Psi }_q(\frac{z}{L}) \right] ~\left[ \textrm{ln}\left( \frac{z}{z_{0}} \right) - {\Psi }_m(\frac{z}{L}) \right] }~, \end{aligned}$$

where $\kappa =0.4$ is the von Kármán constant, $z_{0q}$ and $z_0$ (m) are the roughness lengths for humidity and momentum, respectively, ${\Psi }_q$ and ${\Psi }_m$ are the stability corrections for latent heat and momentum, respectively, *z* is the sampling height, and *L* is the Obukhov length. The momentum and sensible heat fluxes are parametrized using analogue equations (e.g., King et al. 2001).

Based on the friction velocity measured by the ultrasonic anemometer in neutral conditions, $z_0$ was estimated to be $10^{-4}$ m, a typical value for rather flat snow surfaces. This value was assumed to remain constant with varying friction velocity. This assumption is supported by Andreas et al. (2010), at least for friction velocities larger than 0.15 m $\hbox {s}^{-1}$. Friction velocities below this value are very rare in the present dataset. The roughness lengths for humidity and temperature were calculated as a function of the friction velocity and $z_0$ according to the parametrization of Andreas (1987). Although measurement uncertainties make it difficult to validate this parametrization, a couple of studies support the plausibility of the parametrization at snow-covered sites with $z_0 < 10^{-3}$ m (Smeets and van den Broeke 2008; Andreas et al. 2010; Park et al. 2010; Vignon et al. 2017; Liu et al. 2020).

The Monin–Obukhov stability parameter, $z~L^{-1}$, was calculated iteratively, starting with a value of zero. For stable and neutral conditions ($z~L^{-1} \ge 0$), the stability correction of Stearns and Weidner (1993) was used and for unstable conditions ($z~L^{-1} < 0$), the stability correction of Businger et al. (1971) with the modifications of Högström (1987) was used. In the study of Schlögl et al. (2017), the MOST parametrization showed a low sensitivity to the choice of the stability correction functions, when applied to stable conditions at alpine and polar sites.

#### Drifting-Snow Mass Flux

The SPC optically detects snow particles in a volume (width $\times$ height $\times$ depth) of 25 $\times$ 2 $\times$ 0.5 $\hbox {mm}^3$ (Sugiura et al. 1998). The instrument aligns itself with the wind direction by means of a wind vane. The measurement output includes particle numbers for 64 size classes with mean diameters from $36~\upmu \textrm{m}$ to $490~\upmu \textrm{m}$ and time intervals of 1 s. The recorded particle diameters are equivalent diameters based on the assumption of a spherical shape. Because the measurement volume is limited by a depth of $500~\upmu \textrm{m}$, particles with larger diameters are assigned to the largest size class. The SPC cannot distinguish between particles lifted from the surface and snowfall particles but snowfall can be ruled out if the MRR only detects particles below a certain height.

Because the measured particle diameters depend on temperature, the SPC also records the temperature to enable a correction for the difference between the observed temperature and the reference temperature during factory calibration. This temperature correction was performed using the post-processing software of the manufacturer and increased the particle sizes because the observed temperatures were higher than the reference temperature. The horizontal mass flux of drifting or blowing snow ($\text {kg m}^{-2}~\text {min}^{-1}$) was calculated as described by Sugiura et al. (1998), assuming spherical snow particles with the density of ice, $918.4~\text {kg m}^{-3}$.

#### Estimating the Depth of the Blowing-Snow Layer

The depth of the blowing-snow layer was estimated using two standard products provided by the MRR instrument: the signal-to-noise ratio (*SNR*) and the radial velocity component. The signal-to-noise ratio (dB) is defined by

4 $$\begin{aligned} SNR = 10 \, \log _{10}{\frac{S}{N}} = 10 \, \log _{10}{\frac{\int _{v_{r1}}^{v_{r2}} (s(v)-s_n) \textrm{d}v_r}{s_n\,(v_{r2}-v_{r1})}}~, \end{aligned}$$

where *S* and *N* are signal power and noise power, respectively, *s* is the spectral signal power of the raw Doppler spectrum, $s_n$ is the value for the noise level, and $v_{r1}$ and $v_{r2}$ are the radial velocity components limiting the spectral peak. With the tilted MRR configuration, the horizontal projection of the radial velocity component ($\text {m s}^{-1}$),

5 $$\begin{aligned} u_{MRR} = \cos (\beta )~v_r~, \end{aligned}$$

is a measure of the mean speed of blowing-snow particles, which is close to the wind speed. Here, $\beta = 7^\circ$ is the elevation angle of the radar beam and $v_r~(\text {m s}^{-1})$ is the radial velocity component.

**Fig. 2:**
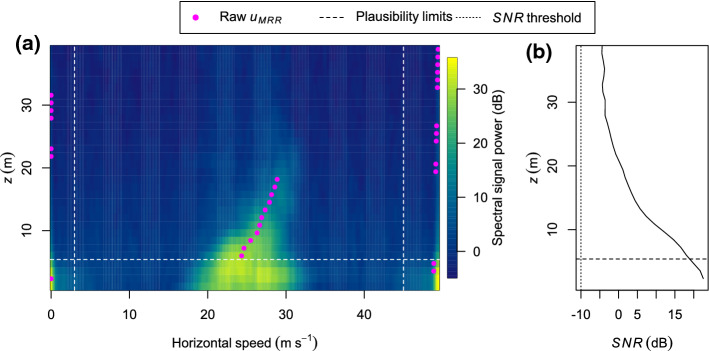
Example raw data of the Micro Rain Radar (2-s averages, 11 January 2019, 1301 UTC): **a** Doppler spectrum and horizontal projection of the radial velocity component ($u_{MRR}$), **b** signal-to-noise ratio (*SNR*) as a function of height (*z*)

The raw Doppler spectra of the MRR frequently contained artefacts, characterized by a high spectral signal power at radial speeds close to the minimum (0 $\text {m s}^{-1}$) and maximum (48 $\text {m s}^{-1}$) values and mainly in the lowest few range gates (Fig. 2a). Possible reasons are ground clutter and enhanced turbulence around the offset parabolic dish resulting in radial speeds around zero, which partly appear at the upper edge of the velocity spectrum because of aliasing. Due to these artefacts, the first four range gates were discarded. Thus, our estimate of the depth of the blowing-snow layer only accounts for layers that exceed a height of 5.4 m. Raw data of both $u_{MRR}$ and *SNR* can be affected by the artefacts, especially at low heights and with an absent or weak blowing-snow signal. The influence of the artefacts was mostly evident from implausible radial speeds that are either close to zero or 48 $\text {m s}^{-1}$. Thus, $u_{MRR}$ values below 3 $\text {m s}^{-1}$ or above 45 $\text {m s}^{-1}$ were discarded.

Before averaging the data per 10-min intervals, the *SNR* was converted from dB units to the dimensionless power ratio. After the averaging, the power ratio was converted back to dB units. The depth of the blowing-snow layer was estimated to be the height up to which (i) the average *SNR* was larger than $-10$ dB and (ii) the instantaneous $u_{MRR}$ values were at least partly between the plausibility limits mentioned above. For several points in time, this estimate was validated by visually inspecting the raw and the 10-min mean Doppler spectra (Fig. 2).

### Simulations

The LES–LSM model is able to capture the effect of turbulent variations in space and time on the interaction between individual drifting/blowing-snow particles and the atmosphere. The simulation set-up aimed at reproducing the steady-state meteorological conditions measured during a 10-min interval. With a height of 6 m, the simulation domain comprised the near-surface atmosphere above a horizontal snow surface with an area of 18 $\times$ 18 $\hbox {m}^2$. The domain contained 96 and 64 grid points along the horizontal and vertical directions, respectively. The grid spacing was uniform in the horizontal directions (0.1875 m) and stretched in the vertical direction (from 0.015 m at the surface to 0.172 m at the upper boundary). The domain size and grid spacing was a trade-off between an acceptable computation time, a high spatial resolution, and large (horizontal) dimensions that enable the development of large-scale coherent structures (Munters et al. 2016). High spatial and temporal resolutions are required to resolve the motion of drifting/blowing-snow particles in a Lagrangian frame of reference and the thermodynamic interaction with the air. For this reason, a timestep of $5 \times 10^{-5}$ s was used. A disadvantage of the rather small domain size is the fact that the maximum size of represented turbulence elements is limited and the contribution of larger turbulence elements to the turbulent fluxes is missing in the simulations. This limitation is currently unavoidable because a larger domain size would lead to unacceptably long simulation times.

In the simulations, the flow is driven by a constant large-scale pressure gradient. The turbulent airflow is simulated by solving the Navier–Stokes equations for incompressible flows while the fields of air temperature and specific humidity are computed using advection–diffusion equations. Subgrid-scale turbulent fluxes are taken into account by applying the scale-dependent Lagrangian dynamic model of Bou-Zeid et al. (2005). Snow transport is simulated considering drag and gravitational forces. To improve the computation time, the trajectories and properties of the snow particles are not modelled individually but for groups of identical particles. Within each group, the particles are assumed to have the same trajectory and identical properties such as location, diameter, mass, and temperature. The number of particles in a group can range between 5000 and 250,000 and is determined during aerodynamic or splash entrainment depending on the local surface shear stress or the impact properties of a group of particles, respectively. After reaching a steady state, the total number of particles aloft was approximately $9.16 \times 10^8$ and the number of particle groups was approximately 38,500. Aerodynamic entrainment, rebound, and ejection of particles at the surface are simulated through statistical formulae based on conservation principles (Comola and Lehning 2017; supplement of Sharma et al. 2018).

**Fig. 3:**
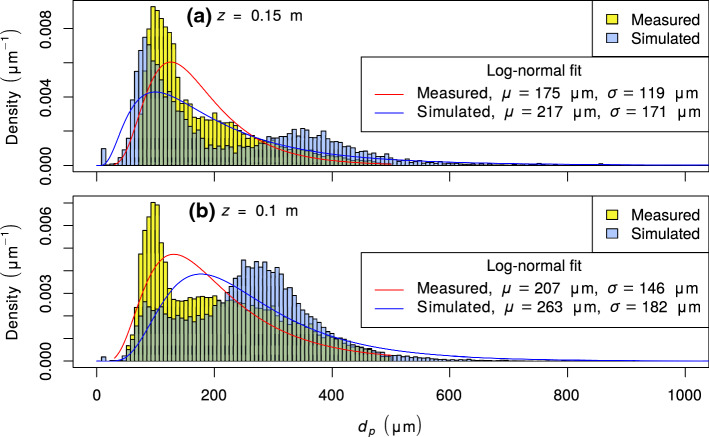
Particle size distributions for the Drift_1 simulation set-up and SPC measurements at the S17 site on 11 January 2019, 0040 to 0050 UTC: probability density as a function of particle diameter ($d_p$) at a height of **a** 0.15 m and **b** 0.1 m. The legend specifies the mean ($\mu$) and the standard deviation ($\sigma$) of $d_p$ for a log-normal fit based on maximum-likelihood estimation

A summary of the simulation parameters is presented in Table 2. Two cases were simulated: a case with significant snow transport named ‘Drift’ and another case with negligible snow transport named ‘NoDrift’. For each case, the simulation was repeated once, using modified values for the upper boundary conditions for humidity and temperature to study the sensitivity of the modelled sublimation. In the following, the four set-ups are referred to as Drift_1, Drift_2, NoDrift_1, and NoDrift_2. The initial particle diameter of entrained particles is taken from a log-normal distribution that results in similar sizes of drifting/blowing-snow particles compared to the SPC measurements (Fig. 3). The choice of a log-normal distribution is based on the study of Colbeck (1986), which determined a log-normal size distribution in water-saturated snow. The initial particle temperature is equal to the surface temperature.

**Table 2:** Important simulation parameters: two cases were simulated (Drift, NoDrift) and for each case, the simulation was repeated with a modified upper boundary condition. For the NoDrift case, the parameters are only specified if they differ from those of the Drift case

Parameter	Symbol	Units	Drift	NoDrift
Specific heat capacity of ice	$c_i$	$\text {J kg}^{-1}~\text {K}^{-1}$	2035.7	
Specific heat capacity of air	$c_{p,f}$	$\text {J kg}^{-1}~\text {K}^{-1}$	1005.0	
Specific heat capacity of vapour	$c_{p,v}$	$\text {J kg}^{-1}~\text {K}^{-1}$	1854.2	
Diffusivity of vapour in air	*D*	$\text {m}^{2}~\text {s}^{-1}$	$1.96 \times 10^{-5}$	
Thermal conductivity of air	*k*	$\text {W m}^{-1}~\text {K}^{-1}$	0.023	
Density of ice	$\rho _p$	$\text {kg m}^{-3}$	918.4	
Density of air	$\rho$	$\text {kg m}^{-3}$	1.18	
Mean initial particle diameter	${\bar{d}}_p$	$\upmu \textrm{m}$	260	
Standard deviation of initial particle diameter	$\sigma (d_p)$	$\upmu \textrm{m}$	130	
Large-scale pressure-gradient force	$F_x$	m $\hbox {s}^{-2}$	0.068	0.0019
Aerodynamic roughness length	$z_0$	m	$10^{-4}$	
Thermal roughness length	$z_{0T}$	m	$5 \times 10^{-5}$	$10^{-4}$
Humidity roughness length	$z_{0q}$	m	$5 \times 10^{-5}$	$1.2 \times 10^{-4}$
Surface temperature	$T_s$	$^{\circ }$C	$-5.63$	$-2.53$
Surface specific humidity	$q_s$	$\text {g kg}^{-1}$	2.613	3.361
Temperature gradient at upper boundary	d*T* (d$$z)^{-1}$$	K $$\hbox {m}^{-1}$$	12.57	$$-5.76^\textrm{c}$$, $$0^\textrm{d}$$
Specific humidity gradient at upper boundary	d*q* (d$$z)^{-1}$$	$$\text {g kg}^{-1}~\textrm{m}^{-1}$$	$$-1.4^\textrm{a}$$, $$-3.5^\textrm{b}$$	$$-2.280^\textrm{c}$$, $$-0.608^\textrm{d}$$

$$^\textrm{a}$$Drift_1

$$^\textrm{b}$$Drift_2

$$^\textrm{c}$$NoDrift_1

$$^\textrm{d}$$NoDrift_2

The exchange of moisture, heat, and momentum between particles and the atmosphere is represented by source or sink terms in the advection–diffusion and Navier–Stokes equations. To this end, the coupled mass-balance and energy-balance equations for individual snow particles in turbulent flow are solved, neglecting radiative heat transfer (Sharma et al. 2018)

6 $$\begin{aligned} \frac{\textrm{d}m_p}{\textrm{d}t}= & {} \uppi \; D \; d_p \; (\rho _{v,\infty }-\rho _{v,p}(T_p)) \; Sh \;, \end{aligned}$$

7 $$\begin{aligned} c_{i} m_p \frac{\textrm{d}T_p}{\textrm{d}t}= & {} L \frac{\textrm{d}m_p}{\textrm{d}t} + \uppi \; k \; d_p \; (T_{a,\infty }-T_p) \; Nu \;, \end{aligned}$$

where $m_p~(\textrm{kg})$, $d_p~(\textrm{m})$, and $T_p~(\textrm{K})$ are the mass, diameter, and temperature of a spherical snow particle, respectively; $\rho _{v,\infty }~(\text {kg m}^{-3})$ and $T_{a,\infty }~(\textrm{K})$ are the temperature and vapour density of the surrounding air, respectively; $\rho _{v,p}~(\text {kg m}^{-3})$ is the vapour density at the particle surface; *t* (s) is time; *Nu* and *Sh* are the dimensionless Nusselt and Sherwood numbers, respectively; and the remaining parameters are included and explained in Table 2. In contrast to the widely used formula of Thorpe and Mason (1966), Eqs. 6 and 7 account for the temperature difference between the particles and the air. This effect is important for an accurate simulation of the moisture exchange, especially for particles in saltation due to their short residence time in the atmosphere (Sharma et al. 2018).

Periodic boundary conditions are required in the horizontal directions because of a Fourier-based pseudo-spectral approach for computing horizontal gradients (Albertson and Parlange 1999). The lower boundary conditions include constant values for temperature and specific humidity based on the measured average surface temperature and the assumption of saturation at the surface (Table 2). As a result, the surface *LE* and *H* values are given by the subgrid-scale fluxes at the lower boundary, which are calculated by applying the MOST parametrization to the layer between the surface and the next higher grid level (0.0075 m). The surface shear stress is computed in a similar way. In these computations, the same roughness lengths for momentum, temperature, and humidity are used as in the calculation of the measured MOST-based fluxes (Table 2). To achieve steady-state humidity and temperature profiles, constant non-zero gradients of humidity and temperature were prescribed as an upper boundary condition. These gradients resulted in moisture and heat transport through the upper boundary by means of subgrid-scale fluxes while the resolved fluxes were zero due to the condition of zero vertical velocity component at the upper boundary. Therefore, unrealistically strong temperature and humidity gradients were needed at the upper boundary. In an ideal simulation set-up, the domain height would be larger and would correspond to the boundary-layer height. This was not possible due to long simulation times. Nevertheless, the analysis focuses on the lowest 2 m of the domain, which is sufficiently far from the upper boundary to achieve realistic conditions. A few values for the humidity and temperature gradients at the upper boundary were tested until the resulting quasi-stationary profiles agreed with the average in situ measurements.

The initial conditions included linear profiles for temperature and specific humidity and a logarithmic profile for the wind speed. Each simulation covered a period of 850 s. During the first 25 s of the simulations, a stationary turbulent flow developed while snow transport was disabled. Subsequently, snow transport was allowed and in the Drift simulations, the horizontal mass flux reached a steady state after a total of 250 s. At the same time, the vertical profiles of temperature and specific humidity approximately reached a steady state. The subsequent 600 s of the simulations were compared with the measurements.

The simulation data was post-processed to obtain the fluxes *LE* and *H* as a function of height. Due to mass and heat conservation, these fluxes are equal to the mean cumulative exchange of latent and sensible heat between the snow (surface and airborne particles) and the air in steady-state conditions

8 $$\begin{aligned} LE(z)= & {} \frac{1}{N_i} \sum _{i=1}^{N_i} \left[ LE(0) - \frac{1}{A} \sum _{p=1}^{N_p(z)} \left( L n_p \frac{\textrm{d} m_p}{\textrm{d} t} \right) \right] ~, \end{aligned}$$

9 $$\begin{aligned} H(z)= & {} \frac{1}{N_i} \sum _{i=1}^{N_i} \left[ H(0) + \frac{1}{A} \sum _{p=1}^{N_p(z)} \left( n_p \; (S_{h1} + S_{h2}) \right) \right] ~, \end{aligned}$$

where $N_i$ is the number of output times during the steady-state period, *LE*(0) ($\text {W m}^{-2}$) and *H*(0) ($\text {W m}^{-2}$) are the respective surface fluxes (i.e., the fluxes at a height of 0 m corresponding to the surface of the snowpack), $N_p$ is the number of particle groups below height *z*, $n_p$ is the number of particles within group *p*, *A* ($\mathrm {m^2}$) is the area of a horizontal cross-section of the domain, and $S_{h1}$ (W) is the sensible-heat source resulting from convective heat transfer between a particle and the air

10 $$\begin{aligned} S_{h1} = - \uppi \; k \; d_p \; (T_{a,\infty }-T_p) \; Nu~, \end{aligned}$$

where *k* is the thermal conductivity of air ($\text {W m}^{-1}~\textrm{K}^{-1}$). The variable $S_{h2}$ (W) is the sensible-heat source resulting from the temperature change of the vapour exchanged between the particle and the air

11 $$\begin{aligned} S_{h2} = \frac{\textrm{d} m_p}{\textrm{d} t} \; c_{p,v} \; (T_{a,\infty }-T_p)~, \end{aligned}$$

where $c_{p,v}$ ($\text {J kg}^{-1}~\textrm{K}^{-1}$) is the specific heat capacity of vapour.

For the height of the EC measurements (1.9 m), *LE* and *H* were additionally calculated from the simulation data using the EC method. More precisely, the sum of the resolved flux, $F_{res}$ ($\text {W m}^{-2}$), and the parametrized subgrid-scale flux, $F_{SGS}$ ($\text {W m}^{-2}$), was computed as

12 $$\begin{aligned} F = F_{res} + F_{SGS}~, \end{aligned}$$

where *F* ($\text {W m}^{-2}$) represents either *LE* or *H* and $F_{res}$ is based on the covariance $\overline{w'\rho _v'}$ or $\overline{w'T'}$ (Eq. 1). Here, *F* was averaged horizontally over all grid nodes at the considered height, and $F_{SGS}$ only accounts for 1.5% to 1.8% of the magnitude of *F*.

**Fig. 4:**
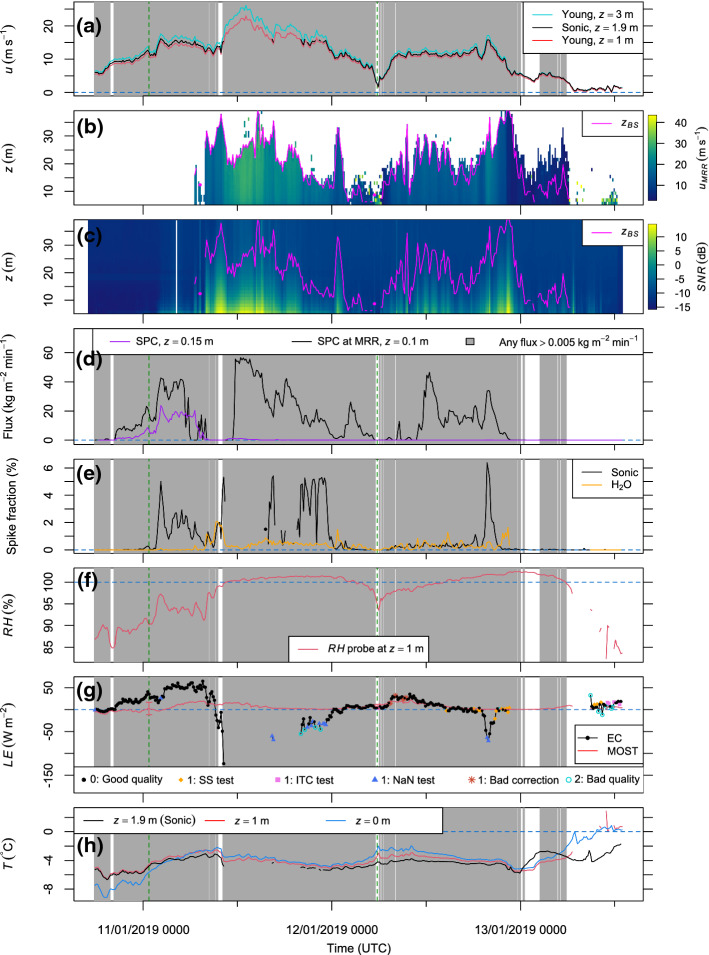
Measurements at the S17 site from 10 to 13 January 2019: 10-min averages of **a** wind speed; **b** horizontal blowing-snow speed ($u_{MRR}$) as a function of height (*z*), where $z_{BS}$ is the depth of the blowing-snow layer; **c** signal-to-noise ratio for the MRR; **d** horizontal flux of drifting and blowing snow based on the SPCs (grey-shaded in all panels if noise is exceeded); **e** fraction of removed spikes in the EC data; **f** relative humidity with respect to ice; **g** latent heat flux for the EC method and the MOST parametrization with quality-control flags and reasons for an intermediate quality, e.g., tests on steady-state (SS) and developed turbulence (or integral turbulence characteristic, ITC); and **h** air and surface temperatures. Instrument-induced standard uncertainties of MOST-based fluxes are shown for two example situations (dashed green), which are compared with simulations in Fig. 6 and Online Resource 4

## Results and Discussion

### Discrepancy Between the Measurement Techniques

From 10 to 13 January 2019, a strong storm with a 10-min averaged wind speed of up to $23~\text {m s}^{-1}$ at a height of 1 m occurred at the S17 site (Fig. 4a). During an initial period with increasing wind speeds, the radial velocity component measured by the MRR was beyond the plausibility limits and was discarded (Fig. 4b). This observation is consistent with low *SNR* values for the MRR signal, indicating that blowing snow did not exceed the minimum detection height of 5.4 m until 11 January 2019, 0630 UTC (Fig. 4c). However, the SPCs measured drifting snow during that initial period with a maximum horizontal mass flux of $38~\text {kg m}^{-2}~\text {min}^{-1}$ and $19~\text {kg m}^{-2}~\textrm{min}^{-1}$ at a height of 0.1 m and 0.15 m, respectively (Fig. 4d). Periods in which at least one of the SPCs indicated drifting snow are highlighted by the grey shading in Fig. 4 using a noise threshold of $0.005~\text {kg m}^{-2}~\text {min}^{-1}$. As of 11 January, 0000 UTC, spikes were removed from the data of the ultrasonic anemometer, suggesting that the blowing-snow layer extended up to a height of 1.9 m or more (Fig. 4e).

At a height of 1 m, relative humidity with respect to ice was initially 87% and had a positive trend before reaching 100% in the late morning of 11 January 2019 (Fig. 4f). During the first three hours of the investigation period both the EC method and the MOST parametrization yield a value of *LE* close to zero, while the drifting-snow mass flux was very low (Fig. 4d, g). Subsequently, the EC method indicates an increasing value of *LE* of up to $65~\text {W m}^{-2}$, coinciding with an increasing trend in the drifting-snow mass flux. Thus, the increase in relative humidity may be largely explained by sublimation of drifting and blowing snow. The maximum sublimation rate was reached before the near-surface air became saturated. For the MOST-based estimate, the sublimation rate remains much lower, with a maximum value of $21~\text {W m}^{-2}$, three times lower than the EC-based value. Initially, the snow surface was cooler than the air, indicating a statically stable stratification (Fig. 4h). After the drifting-snow mass flux reached high values on 11 January 2019, 0000 UTC, the surface temperature increased more rapidly than the air temperature, resulting in an approximately isothermal temperature profile during the rest of the storm event. The strong surface heating may be explained by a combination of strong turbulent mixing, incoming solar radiation, enhanced downward longwave radiation due to drifting and blowing snow, and, potentially, the release of latent heat through vapour deposition at the surface (Yamanouchi and Kawaguchi 1985; Palm et al. 2018).

**Fig. 5:**
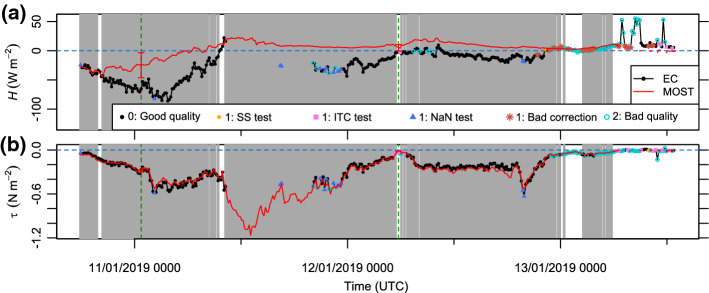
**a** Sensible heat flux and **b** momentum flux for the EC method and the MOST parametrization with quality-control flags, presented in the same way as Fig. 4

When the maximum sublimation rate was reached, the MRR device began to detect a blowing-snow layer with a depth varying between 12 m and 39 m during the subsequent 18 h, which represents the most intense phase of the storm (Fig. 4b, c). During this phase, the SPC at the micrometeorological station measured a mass flux close to zero while the SPC at the MRR site measured high mass fluxes apart from the beginning of the phase (Fig. 4d). In the field, it was observed that drifting-snow particles can form a thin ice layer on the optical windows of the SPCs, especially in warm conditions. This problem likely explains the very low mass fluxes measured by one or both SPCs while blowing snow was evident from the MRR data and from spikes in the EC data (Fig. 4b–e). At the MRR site, the ice layer was manually removed from the SPC windows from time to time but this could not be performed at the micrometeorological station because of its remoteness under such conditions. Apart from this problem, very intense drifting-snow events can result in a saturation of the SPC measurements, i.e., an individual detection of particles is not possible because the particle concentration or velocity is too high.

During the most intense phase of the storm, relative humidity reached a value of 100% at a height of 1 m and the EC method yielded a downward *LE*, suggesting considerable vapour deposition below the measurement height of 1.9 m (Fig. 4f, g). After measuring a minimum *LE* of $-120~\text {W m}^{-2}$, the EC method failed during the highest wind speeds ($>15~\text {m s}^{-1}$) due to data gaps and many artefacts. When the wind speed decreased again, the EC-based *LE* values still indicate considerable vapour deposition with values increasing gradually from $-70~\text {W m}^{-2}$ to zero. For a few data points, the quality-control tests indicate an intermediate or bad quality, mainly due to a large fraction of missing data. Because the depth of the blowing-snow layer was much higher than the sensor heights for the in situ measurements, it is expected that the vapour deposition in the near-surface atmosphere was offset or outweighed by blowing-snow sublimation in a potentially unsaturated upper part of the blowing-snow layer. In contrast to the EC method, the MOST parametrization does not suggest vapour deposition but a small upward or zero *LE* value while the air was saturated (Fig. 4g).

In the morning of 12 January 2019, the storm ceased for a short time and neither the MRR, nor the SPCs, nor the spike-removal algorithm indicate drifting or blowing snow (Fig. 4a–e). At the same time, the relative humidity dropped to 94% (Fig. 4f). Subsequently, the wind speed increased again and stayed at $11~\text {m s}^{-1}$ for many hours, resulting in drifting and blowing snow. During this phase, relative humidity slowly increased and reached 100% after a few hours. The EC method and the MOST parametrization indicated similar sublimation rates during this phase. The only exception is a short period around 12 January 2019, 2000 UTC, when considerable vapour deposition with an absolute magnitude of up to $70~\text {W m}^{-2}$ was measured by the EC method while the MOST-based estimate is approximately zero. This mismatch coincides with peaks in the wind speed ($15~\text {m s}^{-1}$), in the depth of the blowing-snow layer (36 m), in the drifting-snow mass flux at a height of 0.1 m ($30~\text {kg m}^{-2}~\textrm{min}^{-1}$), and in the spike percentage in the sonic data (6%).

Similar to the *LE* values, the *H* values differed significantly between the measurement methods during most of the time with a considerable amount of drifting and blowing snow (Fig. 5a). In contrast, both methods agree well with respect to the momentum flux during the entire investigation period (Fig. 5b). The quality-control tests for the EC method suggest that the requirements for steady state and well-developed turbulence are fulfilled most of the time.

The discrepancy between the two estimates for *LE* and *H* indicates that drifting and blowing snow can cause significant errors for at least one of the methods. Uncertainties in the MOST-based estimates mainly arise from (i) instrument uncertainties and (ii) heat and moisture sources or sinks above the surface, violating the assumption of height-constant turbulent fluxes. Here, effect (i) is discussed whereas effect (ii) is discussed later. The relative error caused by effect (i) increases with the wind speed because strong turbulent mixing results in relatively small vertical differences in temperature and humidity, compared with their instrument uncertainties. Instrument-induced uncertainties in the fluxes were estimated by means of uncertainty propagation for two example situations with high and low wind speeds, respectively (dashed green lines in Figs. 4, 5) (Joint Committee for Guides in Metrology 2008). This estimation accounts for instrument uncertainties in air and surface temperatures, relative humidity, and wind speed, while uncertainties in other parameters such as roughness length and Obukhov length are assumed to be negligible. For the fluxes *LE* and *H*, the combined standard uncertainties are considerable, mostly due to large uncertainties in air temperature and relative humidity, whereas the momentum flux is little affected by instrument uncertainties (Table 3, Fig. 4g, 5a, 5b). In the example situation with a high wind speed and significant snow transport, the instrument uncertainties only explain approximately half of the difference between the measurement methods for *LE* and *H*, which suggests that other sources of uncertainty are important as well. In the example situation with a low wind speed and without snow transport, the instrument-induced uncertainty in *LE* is larger than the difference between the measurement methods and the instrument-induced uncertainty in *H* accounts for 68% of the difference between the methods.

**Table 3:** Instrument-induced uncertainties for some measurements and for the resulting Monin–Obukhov bulk fluxes in a situation with a high wind speed (11 January 2019, 0040 to 0050 UTC) and a situation with a low wind speed (12 January 2019 0540 to 0550 UTC)

Variable	Units	Standard uncertainty	
		11/01/2019 0045	12/01/2019 0545
Air temperature	$^\circ \textrm{C}$	0.9	0.9
Surface temperature	$^\circ \textrm{C}$	0.2	0.2
Relative humidity	%	4	6$^\textrm{a}$
Wind speed	$\text {m s}^{-1}$	0.3	0.3
Latent heat flux (bulk)	$\text {W m}^{-2}$	14	5
Sensible heat flux (bulk)	$\text {W m}^{-2}$	21	6
Momentum flux (bulk)	$\text {N m}^{-2}$	0.015	0.003

$^\textrm{a}$Increased uncertainty due to a relative humidity $>90\%$

For the EC method, the largest problems and uncertainties arise from data gaps and spikes in the high-frequency data during periods with very high wind speeds. The choice of the threshold in the spike-removal algorithm remains subjective and an influence of undetected, less obvious spikes cannot be excluded. However, as long as the mass flux of blowing snow is rather weak at the height of the EC instruments, enough valid data records are obtained and the EC method is expected to be more reliable than the MOST parametrization.

### Comparing Measurements and Simulations in a Case with Saltation-Dominated Snow Transport

The LES–LSM set-ups named Drift_1 and Drift_2 are used to gain further insights into the example situation on 11 January 2019, 0040 to 0050 UTC, indicated by a vertical dashed green line in Fig. 4. At that time, both SPCs measured plausible snow mass fluxes of an intermediate magnitude (Fig. 4d). The layer of drifting and blowing snow was too shallow to be detected by the MRR (Fig. 4b, c). Almost no spikes were detected in the EC data, which suggests that the vast majority of snow particles was transported below the measurement height of 1.9 m (Fig. 4e). Thus, the EC measurements are deemed reliable. The main purpose of the simulations was to evaluate whether the simulated sublimation rates are in good agreement with the EC measurements, thereby increasing the confidence in both the measurements and the simulations. Additionally, the simulations are used to investigate the vertical thermodynamic structure of the near-surface atmosphere and to estimate the error of the MOST parametrization without the influence of instrument uncertainties.

**Fig. 6:**
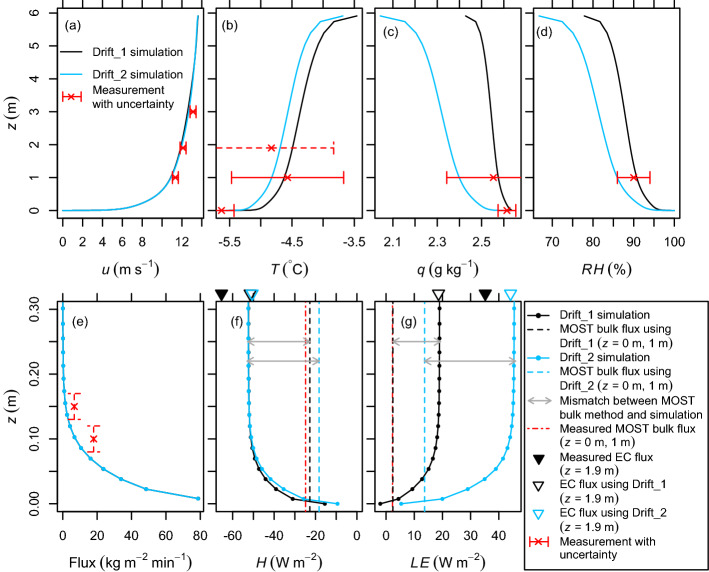
Average vertical profiles for a quasi-stationary 10-min period in two LES–LSM set-ups (Drift_1, Drift_2) with a different humidity gradient at the upper boundary: **a** Wind speed, **b** temperature, **c** specific humidity, **d** relative humidity with respect to ice, **e** horizontal snow mass flux, **f** cumulative sensible heat exchange, **g** cumulative latent heat exchange. For comparison, 10-min averaged measurements from the S17 site (11 January 2019, 0040 to 0050 UTC) are shown with instrument-specific standard uncertainties (dashed if estimated). Note that **e**–**g** only show the lowest 0.3 m of the profile

The simulations reproduced the measured wind speeds well (Fig. 6a). Most of the simulated snow transport occurred in the saltation layer (approximately the lowest 0.1 m) and the simulated horizontal snow mass flux was almost zero above a height of 0.2 m (Fig. 6e). Compared with the SPC measurements, the snow mass flux is somewhat underestimated. Possible reasons for this underestimation are shortcomings in the simulation, uncertainties in the SPC height due to spatial and temporal variations in surface elevation, uncertainties in the measured particle numbers, and uncertainties in the assumed particle density and shape. Figure 6e only shows an estimate for the uncertainty in the SPC height (dashed vertical bars) because the other uncertainties are difficult to quantify. For example, a non-spherical particle, especially if it is strongly anisotropic, can contribute to an overestimation or underestimation of the mass flux depending on the particle’s orientation because the projected area can be larger or smaller than for a spherical particle of the same mass. Whether this effect leads to an overestimation or underestimation of the average mass of many particles, may depend on the shape of the particles.

In the Drift_1 simulation, air temperature, specific humidity, and relative humidity are very close to the values measured at a height of 1 m (Fig. 6b–d). In this case, the specific humidity is at a maximum at the height of the first grid node above the surface (0.075 m) due to drifting-snow sublimation, resulting in a negative value of *LE* at the surface ($-2~\text {W m}^{-2}$), i.e., vapour deposition (Fig. 6c, g). The vertical profiles shown in Fig. 6f, g (solid lines) represent the cumulative sensible and latent heat exchange described in Sect. 2.3. Sublimation of drifting and blowing snow cause a significant increase in *LE* with height, at least in the lowest 0.1 m of the domain (Fig. 6g). Above, $LE=19~\text {W m}^{-2}$ for the Drift_1 simulation, which is higher than the measured MOST-based flux ($2~\text {W m}^{-2} \pm 14~\text {W m}^{-2}$) and lower than the measured EC flux ($35~\text {W m}^{-2}$). Here, the uncertainty of $14~\text {W m}^{-2}$ only accounts for instrument-induced uncertainties of the measured MOST-based flux, whereas the theory-related error is discussed later. The uncertainty of the measured EC flux is not known but rather small because snow transport was negligible at the sensor height. Additionally, the quality-control tests indicate good data quality and the assumptions about advection are expected to be largely fulfilled due to the almost flat and homogeneous terrain. The simulated *H* is directed downward with a value of $-53~\text {W m}^{-2}$ above a height of 0.1 m and reduces when approaching the surface because of the heat sink associated with drifting-snow sublimation (Fig. 6f). The simulated *H* value is between the measured values and a bit closer to the EC ($-65~\text {W m}^{-2}$) than the MOST-based measurements ($-25 \pm 21~\text {W m}^{-2}$).

To crosscheck the theory of the EC method and the consistency of the simulation, the *LE* and *H* values based on the simulated covariances and subgrid-scale fluxes at the height of the EC instruments are also shown in Fig. 6f, g (open triangles). As expected, these values are very close to the cumulative exchange of latent and sensible heat, respectively. This finding confirms that the theory of the EC method is applicable to conditions of drifting and blowing snow.

The Drift_2 set-up was used to study the sensitivity of *LE* with respect to a more negative gradient in specific humidity at the upper domain boundary. Although this condition results in a much lower specific humidity, a lower relative humidity, and slightly lower air temperatures, these quantities are still within the uncertainty range (Fig. 6b–d). In the Drift_2 simulation, the surface exhibits the maximum specific humidity and the surface value of *LE* is positive ($5~\text {W m}^{-2}$) (Fig. 6c, g). With a value of $46~\text {W m}^{-2}$ above a height of 0.1 m, the *LE* value is significantly higher than in the Drift_1 simulation and higher than the measured EC flux (Fig. 6g). The value of *H* is approximately the same for both simulations (Fig. 6f), and is underestimated compared with the EC measurements, which could be due to a potential underestimation of the real air temperature and the snow mass flux.

The model-measurement comparison is further complicated by the small dimensions of the model domain, which limit the size of the turbulence structures. Therefore, the simulations are expected to underestimate the turbulent fluxes present in the field. To better understand this effect, the turbulence cospectrum for the vertical wind velocity component and water-vapour density was computed from time series of the Drift_1 simulation. These time series were sampled at a grid point in the horizontal centre of the domain at the same height and sampling frequency as the EC measurements. In contrast to the field measurements, the simulated time series exhibit wave-like trends that are strongly correlated between the variables (Online Resource 1). These trends are due to the fact that large-scale coherent structures are artificially locked in their position in the crosswise direction of the mean flow as a result of periodic horizontal boundary conditions and a short domain length (Munters et al. 2016). This effect is evident from streamwise-oriented bands of increased and reduced wind velocity components, air temperatures, specific humidities, etc. in a time-averaged horizontal cross-section of the domain (Online Resource 2). In the simulation, the location of the large-scale coherent structures varies very slowly in the crosswise direction of the mean flow, resulting in an artificial wave-like trend in the time series (Online Resource 3). In the field, the structures are also expected to be present but they change their crosswise location more quickly than in the simulation. Nevertheless, horizontally- and temporally-averaged quantities in the simulation can still be considered realistic (Munters et al. 2016).

**Fig. 7:**
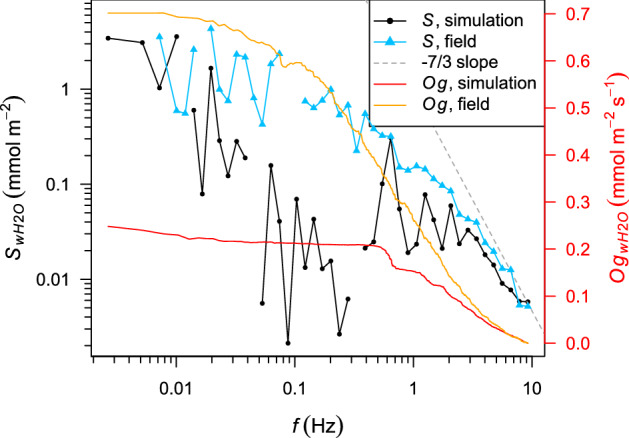
Turbulence cospectra of the vertical wind velocity component (*w*) and molar density of water vapour ($H_2O$) for the Drift_1 simulation set-up and the field measurements (11 January 2019, 0041 to 0048 UTC). Gaps in the bin-averaged spectral density, *S*, are due to negative values, which cannot be represented on the logarithmic scale. The dashed grey line shows the slope of *S* that is typical for the inertial subrange ($S_{wH_{2}O} \propto f^{-7/3}$). The ogive, *Og*, is the integral of spectral density between the considered frequency, *f*, and the Nyquist frequency (10 Hz). The plot is based on time series with $2^{13}$ records, from which a trend was removed. The simulated time series were sampled at a grid point with the same height and at the same frequency as in the field

The trend in the simulated time series would strongly influence the turbulence cospectrum at the lowest frequencies, implying that very large turbulence structures contribute much more to the value of *LE* than in the field. Therefore, the trend was removed using a running mean with a time window of 150 s before computing the cospectrum (Online Resource 1). Figure 7 compares the cospectrum of the vertical wind velocity component and water-vapour density between the Drift_1 simulation and the field measurements.

Although the measured time series does not show a pronounced trend, the same running-mean procedure was applied before computing the cospectrum for the field measurements. For frequencies larger than 0.55 Hz, the spectral density is similar between the simulation results and the field data, indicating a good representation of moisture transport by rather small turbulence structures. However, smaller frequencies between 0.01 Hz and 0.55 Hz are much less pronounced in the simulation compared with the field.

Figure 7 also shows the ogive, which is the integral of the cospectrum between the considered frequency and the Nyquist frequency. In other words, the ogive shows the cummulative contribution of the frequencies to the covariance of the time series. It is important to note that the measured covariance corresponds to the sublimation rate whereas the covariance from one grid point of the simulation domain does not. The reason for the latter is the artificial locking of large-scale coherent structures, resulting in significant vertical advection at a single grid point although the horizontal average of vertical advection is negligible in the Drift simulations. Due to the same effect, the covariance at a single grid point may differ from the horizontally-averaged covariance. Nevertheless, the effect of the limited domain size on the shape of the ogive is expected to be independent from the choice of the sampling point.

For the field, the ogive strongly increases with decreasing frequency from 10 Hz to 0.03 Hz. Consequently, turbulence structures with a wide range in size contribute to the latent heat flux. In contrast, the ogive for the simulation is almost constant for frequencies below 0.55 Hz, indicating that the contribution of this frequency range to the latent heat flux is missing in the simulation. Using Taylor’s frozen-turbulence hypothesis, a frequency of $f = 0.55$ Hz can be translated into a length scale of a turbulence structure of

13 $$\begin{aligned} d = \frac{{\bar{u}}}{f}~, \end{aligned}$$

$= 22~\textrm{m}$ with ${\bar{u}}=12.1~\text {m s}^{-1}$. This length scale is approximately equal to the domain length (18 m). Therefore, turbulent transport associated with larger length scales or smaller frequencies is underestimated in the simulations. At $f = 0.55$ Hz, the ogive for the measurements attains 53% of its maximum, suggesting that the conditions to be reproduced may result in a latent heat flux that is roughly twice as large as suggested by the Drift_1 and Drift_2 simulations. This effect is another possible reason for the fact that the latent and sensible heat fluxes in the Drift_1 simulation have lower absolute magnitudes than those based on the EC measurements. Although a larger model domain is desirable, the computational effort makes it currently impossible because the high spatial and temporal resolutions need to be maintained to properly represent saltation dynamics. Given these considerations, the high sensitivity of the simulated *LE* values to the upper boundary condition for humidity, and the measurement uncertainties, the simulated *LE* and *H* values are in reasonable agreement with the EC measurements.

Apart from that, the simulations demonstrate that the overall latent and sensible heat exchange can be strongly dominated by the contribution of transported snow particles while the surface exchange accounts for a rather small fraction ($-11$% to 12% for *LE* and 18% to 29% for *H* in the analyzed situation, Fig. 6f, g). Similar to the results of Wang et al. (2019), sublimation of drifting and blowing snow is strongest in the air layer of the first grid nodes above the surface (lowest 0.15 m) despite a high relative humidity of 97.1% and 95.6% for the Drift_1 and Drift_2 set-ups, respectively.

### Theory-Related Error in the Monin–Obukhov Bulk Parametrization

By applying the MOST parametrization to the simulated humidity and temperature differences between the surface and a height of 1 m and comparing it with the simulated turbulent fluxes, the theory-related error of the MOST-based measurements can be estimated without an influence of instrument uncertainties. For the Drift_1 and Drift_2 set-ups, the simulation-based Monin–Obukhov bulk flux underestimates the simulated *LE* values by $17~\text {W m}^{-2}$ and $32~\text {W m}^{-2}$ , respectively, corresponding to high relative errors of 87% and 70% (grey arrows in Fig. 6g). For *H*, the mismatch between the simulation-based Monin–Obukhov bulk flux and the simulated flux is $30~\text {W m}^{-2}$ and $35~\text {W m}^{-2}$ based on the Drift_1 and Drift_2 set-ups, respectively, corresponding to relative errors of 57% and 66% (Fig. 6f).

If these mismatches are only due to the theory-related error of the MOST parametrization, they would only exist in conditions of drifting and blowing snow. This hypothesis was tested using the NoDrift simulation set-ups, which reproduced the field conditions observed at the S17 site on 12 January between 0540 UTC and 0550 UTC. During this period, the average wind speed was 2.8 m $\hbox {s}^{-1}$ at a height of 1.9 m and the measured snow mass fluxes were below the noise threshold ($<0.005~\text {kg m}^{-2}~\textrm{min}^{-1}$). In the corresponding simulations, drifting snow was still enabled but few particles are transported and their effect on the turbulent fluxes is negligible. Average vertical profiles for the NoDrift set-ups and a comparison with the field measurements are presented in Online Resource 4. The simulated *LE* values are 8.1 W $\hbox {m}^{-2}$ and 2.0 W $\hbox {m}^{-2}$ for the NoDrift_1 and NoDrift_2 set-ups, respectively. The MOST-based estimates computed from the simulated humidity and temperature differences between the surface and a height of 1 m underestimate this flux by 16% and 20%, respectively. For the sensible heat flux, a similar underestimation of 15% is found for the NoDrift_1 set-up. In the NoDrift_2 simulation, a difference in the sensible heat fluxes is not expected due to an isothermal temperature profile.

The NoDrift simulations show that the theory-related error of the MOST parametrization associated with snow transport is not the only cause for the mismatch between the simulation-based Monin–Obukhov bulk flux and the simulated flux. Another cause may be uncertainties in the simulation. However, the relative underestimation of the fluxes by the MOST parametrization is significantly larger for the Drift set-ups than the NoDrift set-ups. Therefore, a large part of this underestimation can be attributed to the fact that the assumption of height-constant turbulent fluxes is violated in the lowest 0.1 m of the atmosphere due to drifting snow (theory-related error in the MOST parametrization). The moisture sources and heat sinks associated with sublimation of drifting and blowing snow modify the relationship between the temperature and moisture gradients and the turbulent flux, at least in the layer of drifting and blowing snow. For both *LE* and *H*, the sum of the theory-related error and the instrument-induced uncertainty is large enough to explain the difference between the MOST-based and the EC measurements in the investigated case.

In principle, the theory-related error should not occur if MOST was applied to a constant-flux layer above the layer of drifting and blowing snow, i.e., if turbulent fluxes were measured using the profile method. However, Barral et al. (2014) showed that this method is highly sensitive to instrument uncertainties in high wind speeds due to small vertical gradients at typical measurement heights.

Additionally, the LES–LSM results reveal that the MOST parametrization is not a good estimate for surface fluxes during snow-transport events. As expected, the simulation-based Monin–Obukhov bulk estimates are significantly higher than the simulated surface fluxes (Fig. 6f, g): the surface *LE* values are overestimated by 217% and 156% based on the Drift_1 and Drift_2 set-ups, respectively, while the absolute magnitudes of the surface *H* are overestimated by respectively 47% and 95%. The analysis confirms the expectation that the MOST parametrization significantly overestimates the surface fluxes and underestimates the overall latent and sensible heat exchange in typical conditions of drifting and blowing snow. This finding suggests that previous studies such as Thiery et al. (2012) overestimated annual surface sublimation by using MOST-based measurements in both the presence and absence of drifting and blowing snow.

## Conclusions

The present study aimed at better understanding the effect of drifting and blowing snow on the reliability of turbulent-flux estimations, especially *LE*, measured using the MOST parametrization or the EC method. Three days of comprehensive measurements from the S17 site, Antarctica, were discussed and a 10-min interval with saltation-dominated snow transport was reproduced by the LES–LSM model. For the MOST parametrization, the instrument-induced uncertainties in the latent and sensible heat fluxes were quantified using error propagation and the theory-related errors were estimated from the simulation output. The findings verify the hypothesis that the MOST parametrization can be affected by a significant theory-related error in conditions of drifting and blowing snow because the false assumption of height-constant fluxes results in an underestimation of the total latent and sensible heat exchange (surface and drifting/blowing snow) and in an overestimation of the absolute surface fluxes. This error may severely affect estimates of the surface energy and mass balance in experimental and modelling studies employing the MOST parametrization. While the error can be large for instantaneous fluxes, the effect on monthly or yearly averages remains to be explored because an underestimation of both upward and downward fluxes reduces the effect on the average flux. In contrast to the latent and sensible fluxes, the momentum flux measured by the Monin–Obukhov bulk method remains in good agreement with the EC measurements in conditions of drifting and blowing snow. To measure latent and sensible fluxes, the EC method is preferable over the MOST parametrization as long as intense blowing-snow fluxes do not reach the sensor height and result in data gaps. In the case of a shallow layer of drifting and blowing snow, the latent and sensible fluxes based on the EC measurements are of the same order of magnitude as the simulated ones although the simulated latent heat fluxes are sensitive with respect to the upper boundary condition for specific humidity. To improve future model-measurement comparisons, a more accurate sensor for air temperature and relative humidity should be used, which would help to constrain the range of possible upper boundary conditions and simulated fluxes. The plausibility of the EC method is indirectly supported by the fact that the difference between the measurement methods is entirely explained by the errors of the MOST parametrization in the case study with a shallow layer of drifting and blowing snow. However, if blowing snow extends up to the sensor height, it remains unclear whether the removal of spikes from the EC raw data is complete and prevents significant uncertainties. A reliable determination of the moisture exchange between the snow and the atmosphere is not only important for quantifying the surface mass balance but also for understanding changes in the isotopic composition of surface snow. Isotopic fractionation associated with strong sublimation and vapour deposition in a layer of drifting and blowing snow is expected to change the abundance of stable water isotopes in the snow particles and the surface snow. Future studies could investigate a potential effect of these processes on the isotopic signature of ice cores and temperature reconstructions.

### Supplementary Information

Below is the link to the electronic supplementary material.

## Figures and Tables

**Fig. 8:**
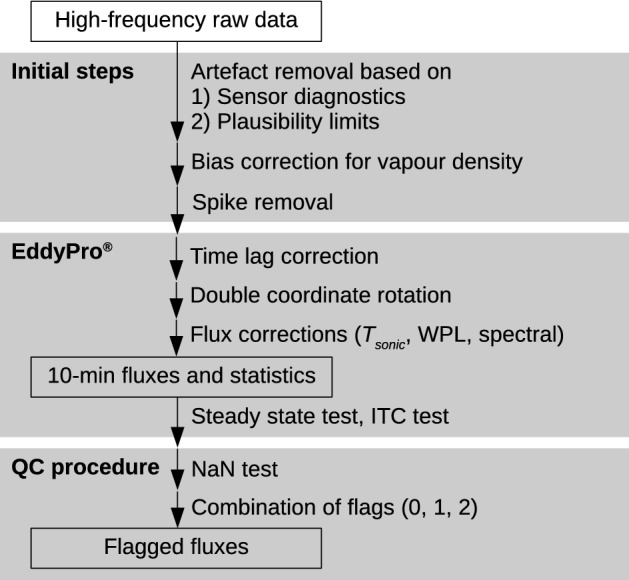
Processing of EC data including initial steps, the use of the EddyPro$^\circledR$ Software, and quality control (QC). The flux corrections account for the difference between sonic temperature ($T_{sonic}$) and air temperature, for density fluctuations (WPL terms), and for spectral losses at high and low frequencies

## Appendix: Processing of Eddy-Covariance Data

The initial steps of the post-processing of the EC data aimed at removing artefacts from the high-frequency data and a bias in the water-vapour density (Fig. 8). Diagnostic flags and values from the ultrasonic anemometer and the gas analyzer were only recorded in aggregated form for each 30-s interval. If the fraction of flags indicating a malfunction of the ultrasonic anemometer is $> 33.3\%$ in the 30-s interval under consideration, the whole interval is discarded in the anemometer data. This threshold is a trade-off between data quality and availability. Because a large number of problematic flags is associated with a large number of spikes in the time series, the remaining artefacts were likely removed later by the spike-removal procedure. The water-vapour density was discarded during the 30-s interval if more than 1% of the records were flagged with a malfunction of the gas analyzer (which was not frequent) or the mean automatic gain control value exceeded 60%, indicating some obstruction of the optical path.

In the next step, lower and upper plausibility limits were applied to the high-frequency records (Table 4). The upper plausibility limit for water-vapour density was based on the saturation value for the upper plausibility limit for sonic temperature, while additionally accounting for a systematic overestimation of the water-vapour density by the gas analyzer.

**Table 4:** Plausibility limits for horizontal (*u*, *v*) and vertical (*w*) wind velocity components, sonic temperature ($T_{sonic}$), and molar density of water vapour ($\rho _{vm}$)

Type of limit	*u*	*v*	*w*	$T_{sonic}$	$\rho _{vm}$
	$(\text {m s}^{-1})$	$(\text {m s}^{-1})$	$(\text {m s}^{-1})$	$(^\circ \textrm{C})$	$(\mathrm {mmol~m^{-3}})$
Lower	$-40$	$-40$	$-10$	$-19$	0
Upper	$+40$	$+40$	$+10$	$+11$	680

This bias was evident from a comparison with low-frequency reference data from a temperature and relative humidity probe. It can be explained by an instrument drift because a zero calibration of the gas analyzer had not been performed for a long time due to the remoteness of the site. Due to a nonlinear calibration curve, a constant bias in the raw absorptance leads to a slightly varying bias in the vapour density and affects the calculation of vapour fluctuations and fluxes (Fratini et al. 2014). Similar to Nieberding et al. (2020), a correction procedure adapted from Fratini et al. (2014) was applied after estimating the bias for each 10-min interval from the difference between the median vapour densities of the gas analyzer and the reference sensor. The bias amounted to approximately $+40~\text {mmol m}^{-3}$ and the correction reduced the magnitude of *LE* by 5%.

Spikes in the high-frequency time series were removed using a novel algorithm. In contrast to existing algorithms, it makes use of the observations that (i) the variables measured by the ultrasonic anemometer often exhibit simultaneous spikes and (ii) the vast majority of spikes are single-point spikes. The algorithm builds on the spike criterion of Mauder et al. (2013)

14 $$\begin{aligned} |d_i| > \frac{q~MAD}{0.6745}~, \end{aligned}$$

where $|d_i|$ is the absolute difference between an instantaneous value and the 5-min median of a time series, *MAD* is median absolute deviation, and $q = 7$ is an empirical factor. The denominator in Eq. 14 relates the *MAD* with the standard deviation if the instantaneous values reflect a normal distribution. Instead of $|d_i|$, we use a metric for single-point spikes taking the two neighbouring data points into account

15 $$\begin{aligned} {\hat{x}}=|d_i| - 0.5(|d_{i-1}|+|d_{i+1}|)~, \end{aligned}$$

where the spike metric ${\hat{x}}$ becomes positive and large for single-point spikes. In this calculation, NaN (not a number) values are treated as being equal to the 5-min median. The spike criterion for the anemometer variables combines the respective spike metrics as follows

16 $$\begin{aligned} \frac{{\hat{u}}}{MAD(u)}+\frac{{\hat{v}}}{MAD(v)}+\frac{{\hat{w}}}{MAD(w)}+\frac{{\hat{T}}_{sonic}}{MAD(T_{sonic})} > \frac{q_{s}}{0.6745}~, \end{aligned}$$

where *u* and *v* are the horizontal velocity components, *w* is the vertical velocity component, $T_{sonic}$ is sonic temperature, and $q_s = 6$ is an empirical factor. The advantage of this multivariate spike criterion is the detection of artefacts with a less pronounced deviation from the 5-min median, if visible in several variables.

The gas-analyzer variables (water vapour and carbon dioxide) usually did not exhibit spikes at the same time. Thus, the spike criterion for water-vapour density was only based on the water-vapour spike metric

17 $$\begin{aligned} \frac{\hat{\rho _v}}{MAD(\rho _v)} > \frac{q_{s}}{0.6745}~, \end{aligned}$$

with the same empirical factor of $q_s = 6$. Spikes were replaced by NaN values and the spike removal was iterated until the last iteration increased the total number of spikes by less than 5% or until a maximum of ten iterations was reached.

The EC data were further processed using the EddyPro$^\circledR$ Software while allowing for a maximum fraction of missing data of 40% per 10-min interval (LI-COR Biosciences 2019). Potential time lags between the wind and vapour measurements were corrected by maximizing the cross-covariance. The median time lag was 0.10 s. In 1% of the cases, this method suggested a time lag beyond the plausibility limits of $\pm 1.00$ s and a default time lag of 0.15 s was assumed. A double coordinate rotation was applied. Fluctuations were computed using block averaging without detrending. The sensible heat flux was corrected for the difference between sonic and air temperature (Van Dijk et al. 2004). The latent heat flux was corrected for density fluctuations considering the so-called WPL terms (Webb et al. 1980). All turbulent fluxes were corrected for spectral losses at high and low frequencies (Massman 2000, 2001; Moncrieff et al. 2004). The spectral correction for high frequencies increased the *LE*, *H*, and $\tau$ values on average by 6.7%, 1.1%, and 0.9%, respectively. The quality-control procedure is described in Sect. 2.2.1.
